# Expression, purification, crystallization, and preliminary X-ray crystallographic studies of the human adiponectin receptors, AdipoR1 and AdipoR2

**DOI:** 10.1007/s10969-014-9192-z

**Published:** 2015-01-10

**Authors:** Hiroaki Tanabe, Kanna Motoyama, Mariko Ikeda, Motoaki Wakiyama, Takaho Terada, Noboru Ohsawa, Toshiaki Hosaka, Masakatsu Hato, Yoshifumi Fujii, Yoshihiro Nakamura, Satoshi Ogasawara, Tomoya Hino, Takeshi Murata, So Iwata, Miki Okada-Iwabu, Masato Iwabu, Kunio Hirata, Yoshiaki Kawano, Masaki Yamamoto, Tomomi Kimura-Someya, Mikako Shirouzu, Toshimasa Yamauchi, Takashi Kadowaki, Shigeyuki Yokoyama

**Affiliations:** 1 RIKEN Systems and Structural Biology Center, 1-7-22 Suehiro-cho, Tsurumi-ku, Yokohama, 230-0045 Japan; 2 Department of Biophysics and Biochemistry and Laboratory of Structural Biology, Graduate School of Science, The University of Tokyo, Hongo, Bunkyo-ku, Tokyo, 113-0033 Japan; 3 Division of Structural and Synthetic Biology, RIKEN Center for Life Science Technologies, 1-7-22 Suehiro-cho, Tsurumi-ku, Yokohama, 230-0045 Japan; 4 RIKEN Structural Biology Laboratory, 1-7-22 Suehiro-cho, Tsurumi-ku, Yokohama, 230-0045 Japan; 5 Department of Cell Biology, Graduate School of Medicine, Kyoto University, Yoshida-Konoe-cho, Sakyo-ku, Kyoto, 606-8501 Japan; 6 JST, Research Acceleration Program, Membrane Protein Crystallography Project, Yoshida-Konoe-cho, Sakyo-ku, Kyoto, 606-8501 Japan; 7 Department of Chemistry, Graduate School of Science, Chiba University, Yayoi-cho, Inage, Chiba, 263-8522 Japan; 8 Division of Molecular Biosciences, Membrane Protein Crystallography Group, Imperial College, London, SW7 2AZ UK; 9 Diamond Light Source, Harwell Science and Innovation Campus, Chilton, Didcot, Oxfordshire, OX11 0DE UK; 10 RIKEN SPring-8 Center, Harima Institute, Kouto, Sayo, Hyogo 679-5148 Japan; 11 Department of Diabetes and Metabolic Diseases, Graduate School of Medicine, The University of Tokyo, Hongo, Bunkyo-ku, Tokyo, 113-0033 Japan; 12 Department of Integrated Molecular Science on Metabolic Diseases, 22nd Century Medical and Research Center, The University of Tokyo, Hongo, Bunkyo-ku, Tokyo, 113-0033 Japan; 13 PRESTO, Japan Science and Technology Agency, Kawaguchi, Saitama 332-0012 Japan; 14 CREST, Japan Science and Technology Agency, Kawaguchi, Saitama 332-0012 Japan

**Keywords:** Membrane protein, Adiponectin receptors AdipoR1 and AdipoR2, Purification, Antibody, Crystallization, Lipidic mesophase

## Abstract

The adiponectin receptors (AdipoR1 and AdipoR2) are membrane proteins with seven transmembrane helices. These receptors regulate glucose and fatty acid metabolism, thereby ameliorating type 2 diabetes. The full-length human AdipoR1 and a series of N-terminally truncated mutants of human AdipoR1 and AdipoR2 were expressed in insect cells. In small-scale size exclusion chromatography, the truncated mutants AdipoR1Δ88 (residues 89–375) and AdipoR2Δ99 (residues 100–386) eluted mostly in the intact monodisperse state, while the others eluted primarily as aggregates. However, gel filtration chromatography of the large-scale preparation of the tag-affinity-purified AdipoR1Δ88 revealed the presence of an excessive amount of the aggregated state over the intact state. Since aggregation due to contaminating nucleic acids may have occurred during the sample concentration step, anion-exchange column chromatography was performed immediately after affinity chromatography, to separate the intact AdipoR1Δ88 from the aggregating species. The separated intact AdipoR1Δ88 did not undergo further aggregation, and was successfully purified to homogeneity by gel filtration chromatography. The purified AdipoR1Δ88 and AdipoR2Δ99 proteins were characterized by thermostability assays with 7-diethylamino-3-(4-maleimidophenyl)-4-methyl coumarin, thin layer chromatography of bound lipids, and surface plasmon resonance analysis of ligand binding, demonstrating their structural integrities. The AdipoR1Δ88 and AdipoR2Δ99 proteins were crystallized with the anti-AdipoR1 monoclonal antibody Fv fragment, by the lipidic mesophase method. X-ray diffraction data sets were obtained at resolutions of 2.8 and 2.4 Å, respectively.

## Introduction

Adiponectin is an anti-diabetic and anti-atherogenic adipokine, and is exclusively expressed in adipose tissue [1–4]. Serum adiponectin levels are significantly reduced in patients with obesity, metabolic syndrome, and type 2 diabetes [5]. We previously reported the expression cloning of complementary DNAs encoding adiponectin receptors (*Adipor*) 1 and 2 [6]. These adiponectin receptors, AdipoR1 and AdipoR2, are key membrane proteins that exert anti-metabolic syndrome effects. Adiponectin accomplishes its biological effects by binding to the AdipoR1 and AdipoR2 receptors. In the liver, both adiponectin receptors mediate the major part of the insulin-sensitizing actions of adiponectin, while AdipoR1 primarily does so in skeletal muscle. AdipoR1 and AdipoR2 regulate glucose and fatty acid metabolism partly via the activation of the AMPK [7–9], Ca^2+^ [10], and PPARα [11, 12] signaling pathways. Interestingly, AdipoR1 and AdipoR2 are predicted to contain seven-transmembrane domains [6], but they are structurally distinct from G-protein coupled receptors (GPCRs) [13]. The adiponectin receptors possess an internal N-terminus and an external C-terminus, which is opposite to the topology of GPCRs. Therefore, AdipoRs are predicted to have unique structures, as compared to those of GPCRs.

Here, we report the expression and purification of the N-terminally truncated human AdipoR1 and AdipoR2 proteins, crystallization of the truncated AdipoR1 and AdipoR2 in complexes with the Fv fragment of an anti-AdipoR1 monoclonal antibody, and their preliminary X-ray crystallographic studies.

## Materials and methods

### Plasmid construction

The *Bgl*II-FLAG-TEV-*Bam*HI-*Eco*RI DNA (5′-GGAAGATCTATGGATTACAAGGACGACGACGATAAGGAAAACCTGTATTTTCAGGGCGGATCCGAATTCCCG-3′) and its complementary DNA were synthesized, annealed together, digested with *Bgl*II and *Eco*RI, and subcloned into the *Bam*HI and *Eco*RI sites of pFastBac1. The resulting plasmid encodes a Flag tag followed by a TEV cleavage site at the N-terminus, and is referred to as pFastBac1-FT hereafter. The cDNAs encoding the full-length human AdipoR1 (residues 1–375) and N-terminally-truncated mutants of AdipoR1 and AdipoR2 (AdipoR1Δ46, residues 47–375; Δ76, 77–375; Δ88, 89–375; Δ101, 102–375; and Δ119, 120–375; AdipoR2Δ58, residues 59–386; Δ87, 88–386; Δ99, 100–386; Δ112, 113–386; and Δ130, 131–386) were amplified by PCR. The PCR products were digested with *Bam*HI and *Xho*I for AdipoR1 and *Eco*RI and *Xho*I for AdipoR2, and then inserted into the pFastBac1-FT vector.

### Protein expression in insect cells

High-titer recombinant baculoviruses were obtained with the Bac-to-Bac Baculovirus Expression System (Invitrogen), according to the manufacturer’s protocol. For large-scale and small-scale production of the recombinant proteins, *Trichoplusia ni* (High Five) cells, at densities of 2 × 10^6^ and 2–5 × 10^6 ^cell/ml, respectively, were infected with the high-titer viral stock at a multiplicity of infection (m.o.i.) of 0.5. Cells were harvested by centrifugation at 42-h post infection, and were washed once with phosphate buffer saline (PBS). Cells were flash-frozen in liquid nitrogen, and stored at −80 °C until use.

### Large-scale membrane preparation

For large-scale preparations of the full-length AdipoR1, AdipoR1Δ88, and AdipoR2Δ99 proteins, frozen cells were thawed in high osmotic buffer [10 mM HEPES–NaOH buffer (pH 7.4) containing 1.0 M NaCl, 10 mM MgCl_2_, 20 mM KCl, and EDTA-free Complete Protease Inhibitor Cocktail (Roche)], and disrupted by Dounce homogenization. The raw membranes were collected by ultracentrifugation at 100,000×*g* for 30 min, and were resuspended in high osmotic buffer. These ultracentrifugation and resuspension steps were repeated four times, to remove the peripheral membrane proteins. Finally, the washed membranes were resuspended in 20 mM HEPES–NaOH buffer (pH 7.4) containing 100 mM NaCl and 10 % (v/v) glycerol, flash-frozen with liquid nitrogen, and stored at −80 °C until use. The membrane proteins were quantified with the *DC* Protein Assay (Bio-Rad), using bovine serum albumin (BSA) as the standard.

### Large-scale protein purification

The purified membranes (20 mg/ml of total membrane proteins) were solubilized with 20 mM HEPES–NaOH buffer (pH 7.4) containing 100 mM NaCl, 10 % (v/v) glycerol, and 1 % (w/v) *n*-dodecyl-β-d-maltoside (DDM, Anatrace), for 1–2 h at 4 °C. The insoluble materials were removed by ultracentrifugation at 100,000×*g* for 1 h. The supernatant was filtered (0.45 μm) and incubated with Anti DYKDDDDK Tag Antibody Beads (Wako) in 20 mM HEPES–NaOH buffer (pH 7.4) containing 300 mM NaCl, 10 % (v/v) glycerol, and 0.5 % (w/v) DDM, at 4 °C with gentle agitation. The beads were washed with thirty column volumes of buffer A [20 mM HEPES–NaOH buffer (pH 7.4), containing 10 % (v/v) glycerol, 0.025 % (w/v) DDM, and 0.0001 % (w/v) cholesteryl-hemi-succinate (CHS, Anatrace)], containing 200 mM NaCl. Then, the adsorbed AdipoR1/AdipoR2 proteins were eluted with five column volumes of buffer A containing 100 or 200 mM NaCl and 0.1 mg/ml DYKDDDDK peptide (Wako). Further purification was performed by the following three methods.

First, the affinity-purified sample was concentrated by ultrafiltration with an Ultra-15 30 K-MWCO filter (Millipore), and then loaded on a HiLoad 16/600 Superdex 200 (GE Healthcare) column equilibrated in buffer A containing 200 mM NaCl. Second, the affinity-purified sample, in 100 mM NaCl, was loaded on a 1-ml HiTrap Q column (GE Healthcare) equilibrated with buffer A containing 100 mM NaCl, and was eluted by a 100–1,000 mM linear NaCl gradient in buffer A. The sample was further purified by SEC on a Superdex 200 10/300 (GE Healthcare) column, in buffer A containing 200 mM NaCl. Third, the affinity-purified sample, in 200 mM NaCl, was loaded on a 1-ml HiTrap Q column equilibrated with buffer A containing 200 mM NaCl, and the flow-through fraction was collected. The polyhistidine-tagged TEV protease was added to the anion-exchange-purified fraction, and incubated with the receptor overnight at 4 °C. The receptor was separated from TEV by adsorption to TALON resin (Clontech), and was further purified by SEC on a Superdex 200 10/300 column in buffer A containing 200 mM NaCl. The purities of the AdipoR1Δ88 and AdipoR2Δ99 proteins were assessed by SDS-PAGE.

### Small-scale SEC analysis

Frozen cells were thawed in the high osmotic buffer, and then sonicated to disrupt the cells. The membranes were collected by ultracentrifugation at 100,000×*g* for 15 min, and were resuspended in 20 mM HEPES–NaOH buffer (pH 7.4) containing 150 mM NaCl, 1 mM EDTA, and 5 mM MgCl_2_. The purified membranes (10 mg/ml of membrane protein) were solubilized with 20 mM HEPES–NaOH buffer (pH 7.4) containing 1 % (w/v) DDM, 150 mM NaCl, 1 mM EDTA, and 5 mM MgCl_2_, for 1 h at 4 °C. The insoluble material was removed by ultracentrifugation at 100,000×*g* for 30 min. The supernatant was incubated with 100 μl anti-FLAG M2 affinity gel (Sigma) in 20 mM HEPES–NaOH buffer (pH 7.4) containing 150 mM NaCl, 1 mM EDTA, 5 mM MgCl_2_, 5 % (v/v) glycerol, and 0.5 % (w/v) DDM, at 4 °C with gentle agitation. The beads were washed with five column volumes of 20 mM HEPES–NaOH buffer (pH 7.4) containing 150 mM NaCl, 1 mM EDTA, 5 mM MgCl_2_, 5 % (v/v) glycerol, and 0.04 % (w/v) DDM, and the adsorbed receptor was eluted with five column volumes of the same buffer containing 0.1 mg/ml FLAG peptide (Sigma). The affinity purified sample was then loaded on a Superdex 200 10/300 column in 20 mM HEPES–NaOH buffer (pH 7.4) containing 200 mM NaCl, 10 % (v/v) glycerol, and 0.04 % (w/v) DDM. Fractions (1 ml) were collected. Portions (5 μl) of the eluates were separated by SDS-PAGE, and transferred to a PVDF membrane. The membranes were blocked in 5 % (w/v) dry milk in TBS-T buffer [Tris-buffered saline, 0.1 % (v/v) Tween-20] at room temperature for 1 h. The blocked membranes were detected with the anti-FLAG M2 monoclonal antibody (Sigma) and the anti-mouse IgG, HRP-linked whole antibody from sheep (GE Healthcare) in TBS-T buffer. The membranes were visualized using Immobilon Western Chemiluminescent HRP Substrate (Millipore), and detected with an LAS3000 imager (Fuji).

### Characterization of the purified N-terminally truncated mutants of AdipoR1 and AdipoR2

The purified AdipoR1Δ88 and AdipoR2Δ99 proteins were analyzed by the following three methods. First, the thermal stabilities of AdipoR1Δ88 and AdipoR2Δ99 were analyzed by the 7-diethylamino-3-(4-maleimidophenyl)-4-methyl coumarin (CPM) assay method [14, 15]. The fluorescence of the CPM dye was measured with a 340-nm excitation filter with a 10-nm bandpass and a 460-nm emission filter with a 35-nm bandpass at 40 °C, on a FUSION α Microplate Reader PerkinElmer. Second, the lipids that co-purified with the AdipoR1Δ88 and AdipoR2Δ99 proteins were analyzed by thin layer chromatography (TLC). The full-length AdipoR1 and AdipoR2, prepared by FLAG affinity and anion-exchange chromatography, were also analyzed for comparison. The proteins were dissolved in chloroform/methanol [2:1 (v/v)], and the bound lipids were extracted from the proteins. The extracted samples were applied to a silica gel 60 TLC plate (Merck Millipore), which was then developed by a solvent system composed of chloroform/methanol/water [65:25:4 (v/v)]. The lipids were visualized with acetic acid/sulfuric acid [1:1 (v/v)], the phosphomolybdic reagent (Pierce), and the ninhydrin reagent (Wako). Third, the ligand-binding activity of AdipoR1Δ88 was measured by surface plasmon resonance (SPR) measurements. The purified AdipoR1Δ88 was reconstituted into liposomes [5 mg/ml egg yolk phosphatidylcholine (PC) (Avanti Polar Lipids Inc.) and 0.05 mg/ml biotinyl-phosphatidylethanolamine (biotinyl-PE) (Avanti Polar Lipids Inc.)], and the reconstituted liposomes were immobilized onto a sensor chip SA. Binding analyses were performed with a range of osmotin concentrations (0.5–8 μM) on a Biacore T200 (GE Healthcare).

### Production of the anti-AdipoR1 monoclonal antibody

All animal experiments described here were approved by the Institutional Animal Care and Use Committee of Kyoto University Graduate School of Medicine. The purified untagged AdipoR1Δ88 was reconstituted into liposomes [5 mg/ml egg yolk PC and 1 mg/ml lipid A (Sigma)]. Female BALB/c mice were immunized five times with 0.1 mg doses of the reconstituted AdipoR1Δ88, at intervals of 10 days. Single-cell suspensions were prepared from the spleens of the immunized mice, and the cells were fused with P3U1 myeloma cells, using the conventional polyethylene glycol (PEG) method [16]. Screening of antibodies was performed by three methods, enzyme-linked immunosorbent assay (ELISA), fluorescence-detection SEC (FSEC), and denatured dot blot assays [17, Ogasawara et al., manuscript in preparation]. For ELISA, the purified AdipoR1Δ88 was reconstituted into liposomes containing biotinyl-PE, and was immobilized on Immobilizer Streptavidin plates (Nunc). High-affinity antibodies that formed stable complexes with the purified AdipoR1Δ88 were selected by FSEC, using the fluorescein-conjugated Fab fragment of an anti-mouse IgG (Jackson), on a Superdex 200 5/150 column (GE Healthcare). Antibodies that recognized the native conformation of AdipoR1Δ88 were selected by dot blot assays with SDS-denatured AdipoR1Δ88. Each selected clone was isolated by the limiting dilution-culture method, and monoclonal hybridoma cell lines producing anti-AdipoR1Δ88 antibodies were established.

### Recombinant production of the Fv fragment of the anti-AdipoR1 antibody

The sequences of the V_H_ and V_L_ regions were determined by the standard method, with total RNA isolated from the hybridoma cells [18]. The cloned V_H_ and V_L_ cDNA fragments were subcloned into the TA-cloning vector, pCR2.1 TOPO (Invitrogen) [19], encoding a fusion protein with an N-terminal His-tag, a SUMO tag, and a SUMO protease cleavage site. The V_H_ and V_L_ fragments were co-synthesized by the *E. coli* cell-free protein synthesis method [20], supplemented with DsbC and the reduced and oxidized forms of glutathione (GSH and GSSG, respectively) to form disulfide bonds [21]. The reaction solution was centrifuged at 20,000×*g* and 4 °C for 10 min. The supernatant was loaded on a 1-ml HisTrap column (GE Healthcare) equilibrated with 20 mM Tris–HCl buffer (pH 8.0) containing 500 mM NaCl and 20 mM imidazole, and was eluted by a 20–500 mM linear gradient of imidazole in 20 mM Tris–HCl buffer (pH 8.0) containing 500 mM NaCl. The His- and SUMO-tags were cleaved by SUMO protease for 2–3 days at room temperature, and were removed by a second passage through the HisTrap column. The protein sample was then loaded on a HiLoad 16/600 Superdex 200 column, equilibrated in 20 mM HEPES–NaOH buffer (pH 7.4) containing 200 mM NaCl. The purified Fv fragment (Fv43) was concentrated to approximately 20 mg/ml, by ultrafiltration with an Ultra-15 10 K-MWCO filter (Millipore).

### Preparation of the AdipoR1·Fv43 and AdipoR2·Fv43 complexes

The Fv43 (24.8 kDa) was mixed with the purified FLAG-tagged AdipoR1Δ88 (35.0 kDa) or untagged AdipoR2Δ99 (32.9 kDa), and incubated on ice for 30 min. The mixture was loaded onto a Superdex 200 10/300 column equilibrated with 20 mM HEPES–NaOH buffer (pH 7.4) containing 200 mM NaCl, 0.025 % (w/v) DDM, and 0.0001 % (w/v) CHS, and was eluted using the same buffer. Fractions containing the complex were collected and concentrated to approximately 15 mg/ml by ultrafiltration (Ultra-4 30 K-MWCO, Millipore). The purities of the AdipoR1Δ88·Fv43 and AdipoR2Δ99·Fv43 complexes were assessed by SDS-PAGE.

### Crystallization and X-ray data collection

The purified AdipoR1Δ88·Fv43 and AdipoR2Δ99·Fv43 complexes were reconstituted into a lipidic mesophase, by mixing with molten lipid in a mechanical syringe mixer [22]. The protein-LCP mixture contained 40 % (w/w) protein solution, 54 % (w/w) monoolein (Sigma) and 6 % (w/w) cholesterol (Sigma). Forty nanoliter drops of the resulting lipidic mesophase sample were dispensed into 96 well glass plates, overlaid with 0.8 μl precipitant solution, and covered with thin cover glasses, by the use of laboratory-constructed manual and robotic micro-dispensers [23, Hato et al., manuscript in preparation]. Crystallization setups were performed at room temperature, and the plates were incubated at 20 °C. Crystals were harvested directly from the lipidic mesophase using MiTeGen micromounts, and flash cooled in liquid nitrogen. Data collection was performed on beamline BL32XU at SPring-8, using an MX225HE CCD detector [24–26]. X-ray diffraction data with a micro beam of 1 μm × 10 μm (horizontal × vertical) were collected at 100 K, by the helical scan method with 1° oscillation. The data from the AdipoR1Δ88·Fv43 and AdipoR2Δ99·Fv43 crystals were indexed, scaled, and merged with the HKL2000 program suite [27] and the XDS package [28], respectively.

## Results and discussion

### Screening of deletion mutants of AdipoR1 and AdipoR2

**Fig. 1:**
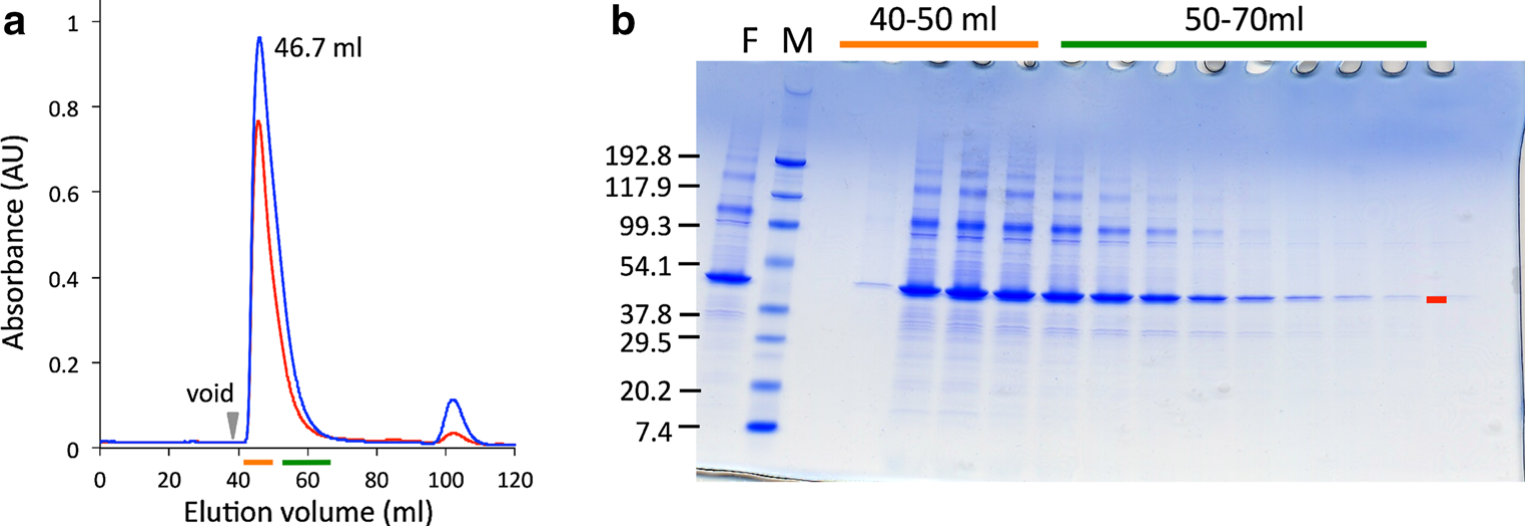
Purification of full-length AdipoR1. **a** Gel filtration chromatogram of the full-length AdipoR1 expressed in High Five insect cells. The proteins were purified by FLAG-affinity chromatography, and then loaded onto a HiLoad 16/600 Superdex 200 column. The main peak retention volume is labeled in *black* (46.7 ml). The absorbances at 280 and 254 nm are colored *blue* and *red*, respectively. **b** SDS-PAGE analysis of the gel-filtration chromatographic fractions containing the full-length AdipoR1. Fractions indicated by the *orange* and *green lines* along the horizontal axis in **a** were analyzed by SDS-PAGE. *Lane F*, the eluate from FLAG-affinity chromatography; *Lane M*, molecular-weight markers (kDa)

The full-length AdipoR1 (residues 1–375) was expressed in baculovirus-infected High Five insect cells, and the membrane fractions were prepared. Upon gel filtration chromatography (size-exclusion chromatography, SEC) on a HiLoad 16/600 Superdex 200 column, the detergent-solubilized full-length AdipoR1 mainly eluted just after the void volume (40 ml) (Fig. 1). If the full-length AdipoR1 was monomeric and monodisperse, then the proteomicelle should exhibit an estimated molecular mass of ca. 125 kDa (the full-length AdipoR1 monomer, 44.7 kDa; DDM micelle, ca. 80 kDa), and elute after 55 ml (the elution volume of ferritin, 440 kDa) on the gel filtration column. However, most of the full-length AdipoR1 eluted before 55 ml (Fig. 1), indicating that this sample was highly polydisperse and not suitable for crystallization.

**Fig. 2:**
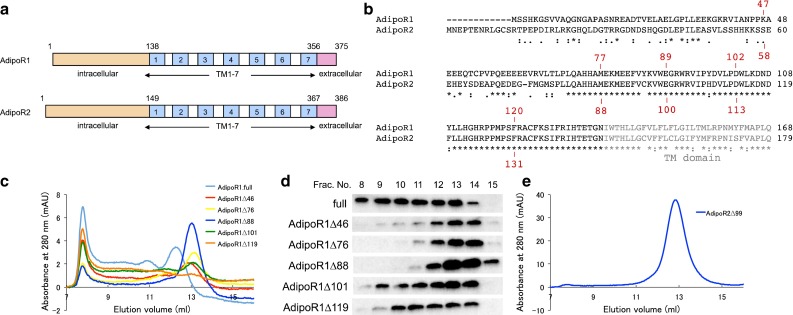
Screening of the N-terminally truncated mutants of AdipoR1 and AdipoR2. Several N-terminal deletion mutants were analyzed by size-exclusion chromatography. **a** Schematic representations of human AdipoR1 (*top*) and AdipoR2 (*bottom*). **b** Amino acid sequences of the N-terminal regions of human AdipoR1 and AdipoR2. The starting amino acid residues of the N-terminally truncated mutants of AdipoR1 and AdipoR2 are numbered in *red*. Amino acids in putative transmembrane domains are shown in *gray letters*. The sequences were aligned with ClustalW [38]. **c** Size-exclusion chromatograms of the full-length form and the N-terminally truncated mutants of AdipoR1. **d** Detection of the AdipoR1 protein in the size-exclusion chromatography fractions by western blotting with the anti-FLAG M2 antibody. **e** Size-exclusion chromatogram of AdipoR2Δ99

We therefore tried to modify the AdipoR1 construct. Prediction servers of protein secondary structure, PSIPRED v3.3 [29], and transmembrane domains, HMMTOP [30], suggested that human AdipoR1 and AdipoR2 have a long N-terminal region, seven transmembrane (TM) helices with short loops connecting the TM helices, and a short C-terminal region (Fig. 2a). Since more than 50 % of the N-terminal region was predicted to be flexible, we speculated that this long N-terminal tail is related to the observed polydispersity of AdipoR1 (Fig. 1).

Therefore, we constructed a series of N-terminally-deleted mutants of AdipoR1 (Fig. 2b). They were expressed on a small scale, and were analyzed by SEC without concentration. In this small-scale SEC analysis of AdipoR1 (Fig. 2c, d), the non-concentrated sample of the full-length AdipoR1 was less aggregated, as compared with the concentrated sample in the large-scale preparation, because it exhibited a small peak eluting at ca. 13 ml (Fig. 1). This ca. 13-ml elution volume in the small-scale SEC analysis should correspond to ca. 65 ml in the large-scale gel filtration chromatography. Deletions of residues 1–46 (Δ46) and 1–76 (Δ76) of AdipoR1 significantly increased the fraction eluting at ca. 13 ml, as compared with the full-length AdipoR1. However, the total amounts of these deletion mutants were much lower than that of the full-length AdipoR1 obtained from the same amount of cells. By contrast, the deletion of residues 1–88 (Δ88) of AdipoR1 further improved the monodispersity in the SEC analysis, and the total amount of this deletion mutant protein was as high as that of the full-length AdipoR1 (Fig. 2c, d). On the other hand, further deletion mutants (AdipoR1Δ101 and AdipoR1Δ119) were less aggregated than the full-length protein, but appreciably more aggregated than AdipoR1Δ88. Thus, we concluded that the AdipoR1Δ88 mutant was the best among the tested deletion mutants. The screening of AdipoR2Δ58, Δ87, Δ99, Δ112, and Δ130 was performed in the same manner, and AdipoR2Δ99 was found to be the best (Fig. 2e). Consequently, the AdipoR1Δ88 and AdipoR2∆99 mutants were selected and used for further crystallization trials.

### Large-scale preparations of N-terminally truncated AdipoR1 and AdipoR2

**Fig. 3:**
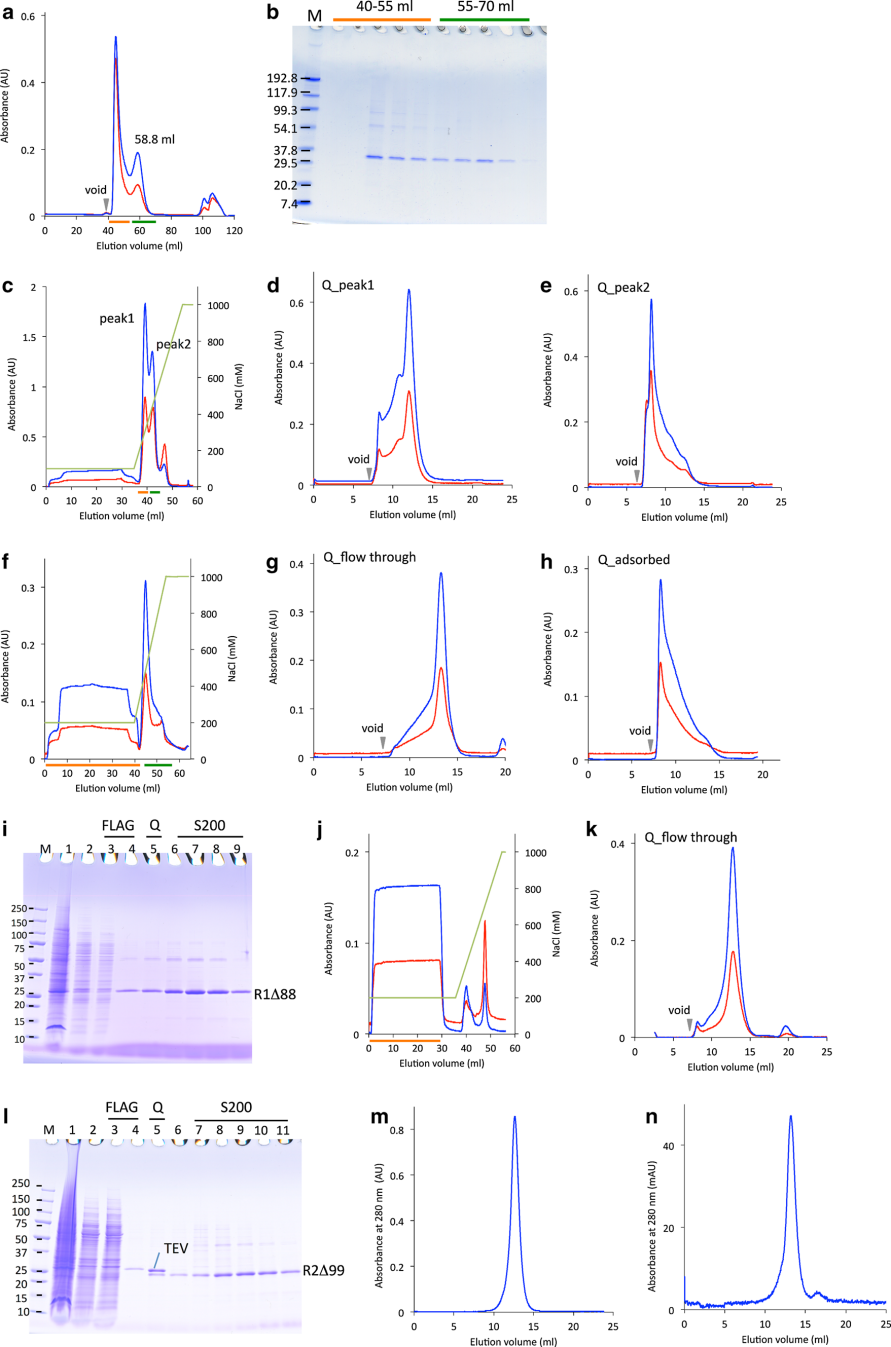
Large-scale preparation of FLAG-tagged AdipoR1Δ88 and AdipoR2Δ99 expressed in High Five cells. In the chromatograms (**a**, **c**–**h**, **j**, **k**, **m**, **n**), the absorbances at 280 and 254 nm, and the NaCl concentration are shown in *blue*, *red*, and *light green*, respectively. **a** Gel filtration chromatogram of FLAG-tagged AdipoR1Δ88 expressed in High Five insect cells. The proteins were purified by FLAG-affinity chromatography, and then chromatographed on a HiLoad 16/600 Superdex 200 column. The main peak elution volume is labeled in *black* (58.8 ml). **b** SDS-PAGE analysis of the gel-filtration chromatographic fractions containing the FLAG-tagged AdipoR1Δ88. Fractions indicated by the *orange* and *green lines* along the horizontal axis in **a** were analyzed by SDS-PAGE. *Lane M*, molecular-weight markers (kDa). **c** Anion-exchange chromatogram of the FLAG-tagged AdipoR1Δ88, with isocratic elution by 100 mM NaCl in buffer A, and subsequently with gradient elution by 100–1,000 mM NaCl in buffer A. **d**, **e** Gel filtration chromatograms of the peak 1 (**d**) and peak 2 (**e**) fractions, indicated by the *orange* and *green lines* in **c**, respectively. **f** Anion-exchange chromatogram of the FLAG-tagged AdipoR1Δ88, with isocratic elution by 200 mM NaCl in buffer A, and subsequently with gradient elution by 200–1,000 mM NaCl in buffer A. **g**, **h** Gel filtration chromatograms of the flow-through (**g**) and adsorbed (**h**) fractions, indicated by the *orange* and *green lines* in **f**, respectively. **i** SDS-PAGE analysis of the FLAG-tagged AdipoR1Δ88. *Lane M*, molecular-weight markers (kDa); *lane 1*, the membrane fraction; *lane 2*, DDM-solubilized membrane proteins in the supernatant after ultracentrifugation; *lane 3*, the flow-through fraction from FLAG-affinity chromatography; *lane 4*, the eluate from FLAG-affinity chromatography; *lane 5*, the flow-through fraction from anion-exchange chromatography (**f**); *lanes 6–9*, the peak fractions of the FLAG-tagged AdipoR1Δ88 from gel filtration chromatography (**g**). **j** Anion-exchange chromatogram of AdipoR2Δ99, with isocratic elution by 200 mM NaCl in buffer A and subsequently with gradient elution by 200–1,000 mM NaCl in buffer A. **k** Gel filtration chromatogram of the flow-through fractions indicated by the *orange line* in **j**. **l** SDS-PAGE analysis of AdipoR2Δ99. *Lane M*, molecular-weight markers (kDa); *lane 1*, the membrane fraction; *lane 2*, DDM-solubilized membrane proteins in the ultracentrifugation supernatant; *lane 3*, the flow-through fraction from FLAG-affinity chromatography; *lane 4*, the eluate from FLAG-affinity chromatography; *lane 5*, the flow-through fraction from anion-exchange chromatography after the TEV protease digestion; *lane 6*, the flow-through fraction from TALON chromatography; *lanes 7–11*, the peak fractions of AdipoR2Δ99 from gel filtration chromatography. **m**, **n** Gel filtration chromatographic analysis of the purified FLAG-tagged AdipoR1Δ88 (**m**) and the purified AdipoR2Δ99 (**n**)

The FLAG-tagged AdipoR1Δ88 and AdipoR2Δ99 were overexpressed in baculovirus-infected High Five insect cells. In general, membrane proteins are purified by minimal steps of chromatography, such as one-step affinity chromatography or two-step (affinity and size-exclusion) chromatography, to avoid deterioration caused by delipidation due to excessive washing with detergents. Therefore, the FLAG-tagged AdipoR1Δ88 was partially purified by stepwise FLAG affinity chromatography, and then fractionated by gel filtration chromatography, in which the AdipoR1Δ88 eluted from the void volume (40 ml) to 70 ml, and formed two peaks (Fig. 3a, b). In this large-scale preparation, the affinity-purified AdipoR1Δ88 behaved differently in the gel filtration (Fig. 3a, b), as compared to the small-scale SEC analysis (Fig. 2c, d). The low molecular mass fraction eluting at 58.8 ml (Fig. 3a) was considered to correspond to the monodisperse fraction of AdipoR1Δ88 eluting at 13 ml in the small-scale SEC analysis. On the other hand, the high molecular mass, aggregated fraction was drastically larger in the large-scale preparation, as compared to the small-scale preparation (Figs. 2c, 3a). Therefore, the affinity-purified AdipoR1Δ88 still aggregated during the sample concentration for gel filtration chromatography, as in the case of the full-length AdipoR1 (Fig. 1), although the properties of AdipoR1 were greatly improved by the N-terminal deletion (Δ88).

Accordingly, we explored the cause of the aggregation. As compared with the monodisperse fraction, the aggregated fraction exhibited a stronger absorbance at 254 nm relative to that at 280 nm (Fig. 3a). Therefore, we hypothesized that nucleic acids contained in the FLAG-affinity-purified AdipoR1Δ88 preparation promoted protein aggregation. To quickly remove the putative nucleic acid contamination, we included an anion-exchange column chromatography step immediately after the FLAG affinity chromatography. The FLAG-tagged AdipoR1Δ88, in buffer A containing 100 or 200 mM NaCl, was applied to a 1-ml HiTrap Q column. The FLAG-tagged AdipoR1Δ88, in buffer A containing 100 mM NaCl, was adsorbed on the column and eluted at low salt concentrations (150–250 mM NaCl) (Fig. 3c–e), whereas that in buffer A containing 200 mM NaCl was eluted in the flow-through fraction (Fig. 3f–i). On the other hand, the aggregated AdipoR1Δ88 was adsorbed on the column with either 100 mM or 200 mM NaCl, and was eluted at high salt concentrations (250–400 mM NaCl) (Fig. 3c, e, f, h). Thus, we could quickly separate the “intact” FLAG-tagged AdipoR1Δ88 from the aggregated AdipoR1Δ88, in buffer A containing 200 mM NaCl, by anion-exchange chromatography (Fig. 3f, g). Once it was purified in this manner, the “intact” FLAG-tagged AdipoR1Δ88 did not undergo the fast aggregation, and was eluted as a symmetrical peak in gel filtration chromatography. In other words, the “rotten apple” (the nucleic acid-aggregated AdipoR1) can be removed quickly by the anion-exchange chromatography, before it “spoils the barrel”. As the cause of the aggregation had been found, we tried another method to reduce it: the sonication of the membrane preparation was also useful for breaking up nucleic acids, and the aggregation of the receptors was significantly reduced during the purification step.

In the same manner, the affinity-purified FLAG-tagged AdipoR2Δ99, in buffer A containing 200 mM NaCl, was eluted in the flow-through fraction in the HiTrap Q column chromatography (Fig. 3j–l). The FLAG tag of AdipoR2Δ99 was removed after the HiTrap Q column chromatography. Thus, the FLAG-tagged AdipoR1Δ88 and the untagged AdipoR2Δ99 were purified to near homogeneity (Fig. 3m, n). Removing the nucleic acids during the membrane preparation and the early stages of purification was essential to obtain larger amounts of the highly homogeneous AdipoR1 and AdipoR2 proteins.

### Characterization of the purified AdipoR1Δ88 and AdipoR2Δ99 proteins

**Fig. 4:**
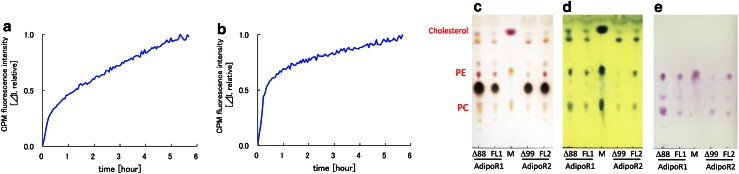
Characterization of the purified AdipoR1Δ88 and AdipoR2Δ99 proteins. **a**, **b** CPM assay of AdipoR1Δ88 (**a**) and AdipoR2Δ99 (**b**). **c**–**e** TLC analysis of AdipoR1Δ88 (Δ88), the full-length AdipoR1 (FL1), AdipoR2Δ99 (Δ99), and the full-length AdipoR2 (FL2). The lipids were visualized with acetic acid/sulfuric acid [1:1 (v/v)] (**c**), the phosphomolybdic reagent (**d**), and the ninhydrin reagent (**e**). *Lane M*, polar lipid mixture (Matreya)

First, the stabilities of the purified AdipoR1Δ88 and AdipoR2Δ99 proteins were analyzed by the CPM assay method [14, 15] (Fig. 4a, b). When the *t*_1/2_ value of thermal denaturation at 40 °C is 17 min or longer, the membrane protein is considered to be sufficiently stable [31]. The corresponding *t*_1/2_ values of our AdipoR1Δ88 and AdipoR2Δ99 proteins are 74 and 20 min, respectively. The CPM profile of AdipoR2Δ99 revealed two phases, fast and slow, probably corresponding to the exposed and transmembrane cysteine residues, respectively, among which the latter reflect the stability of the transmembrane structure. In fact, AdipoR2Δ99 has more exposed cysteine residues than AdipoR1Δ88. Therefore, the *t*_1/2_ value of 20 min for AdipoR2Δ99 may be an underestimate. Consequently, we concluded that both of the present preparations of AdipoR1Δ88 and AdipoR2Δ99 are sufficiently stable. Second, the lipids that co-purified with the AdipoR1Δ88 and AdipoR2Δ99 in these preparations were analyzed by TLC [32] (Fig. 4c–e). Several lipid species co-purified with AdipoR1Δ88 and AdipoR2Δ99, as well as the full-length proteins, indicating that the “bound lipids”, which are important for the native folding of the membrane proteins, are probably retained in these preparations. Third, the purified AdipoR1Δ88 protein was reconstituted into liposomes by the reported method [33]. The reconstituted proteoliposomes exhibited the binding activity for osmotin (*K*_D_ = 0.7 μM; *R*_max_ = 49.2 RU) in the SPR analysis. Together, these results confirmed that we have successfully prepared structurally and functionally intact samples of AdipoR1 and AdipoR2 that are suitable for crystallization.

Thus, crystallization trials of AdipoR1Δ88 and AdipoR2Δ99 were performed with commercially available kits, such as MemStart, MemSys, and MemGold (Molecular Dimensions), by the vapor diffusion method. First, AdipoR2Δ99 crystals were obtained in one of the MemGold kit conditions. Despite extensive attempts to optimize the crystallization conditions to improve the crystal quality, no diffraction was obtained from these crystals. Furthermore, crystallization trials by the lipidic mesophase method were performed for the AdipoR1Δ88 and AdipoR2Δ99 preparations. However, crystals were obtained only in a limited number of conditions, and their diffractions were all poor (data not shown). Consequently, these results indicated that improvement of the crystal packing was necessary.

### Antibody generation

**Fig. 5:**
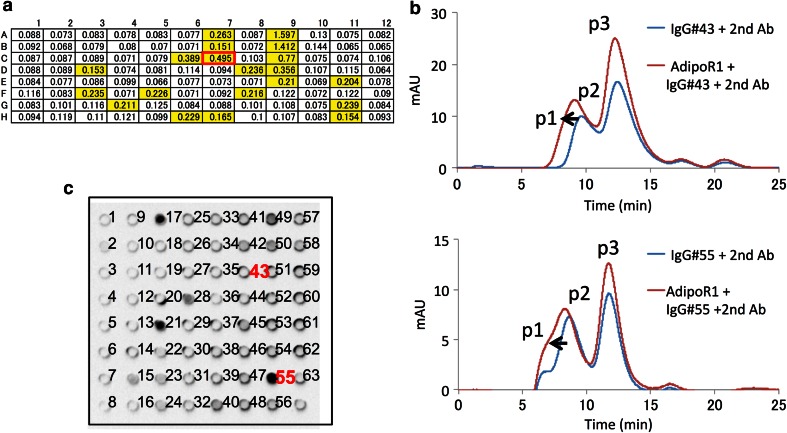
Screening of anti-AdipoR1 antibodies. **a** Representative ELISA plate results. *Yellow areas* represent ELISA positive clones. Well C7, enclosed in a *red box*, indicates clone #43. **b** FSEC analyses of IgG#43 (*top*) and IgG#55 (*bottom*). Peaks *p1*, *p2*, and *p3* represent the peak of the ternary complex (AdipoR1Δ88, the antibody from hybridoma cells, and the fluorescein–Fab fragment), the antibody complex (the antibody from hybridoma cells and the fluorescein–Fab fragment), and the free fluorescein–Fab fragment, respectively. **c** SDS-denatured dot blot screening of anti-AdipoR1 antibodies. IgG#17, IgG#21, and IgG#55 stained denatured AdipoR1. IgG#43 and IgG#55 are numbered in *red*

Antibody fragments are useful tools to improve the resolution in membrane protein crystallography [34]. In fact, GPCRs, such as the β_2_ adrenergic receptor and the A_2A_ adenosine receptor, were co-crystallized with antibody fragments [35, 36]. Therefore, we planned to produce a high affinity and conformational epitope-recognizing anti-AdipoR1 antibody, to improve the crystal packing of AdipoR1. The purified untagged AdipoR1Δ88 was reconstituted into liposomes, and the resultant proteoliposomes were used as the immunogen. Mouse anti-AdipoR1 antibodies were produced by a conventional hybridoma system. Proteoliposomes containing the purified AdipoR1Δ88 and biotinyl-PE were used for screening the antibodies by ELISA (liposome-ELISA). In the first round of liposome-ELISA from 960 wells of hybridoma cultures, 72 positive wells were selected (Fig. 5a). Subsequently, 11 positive clones were selected in the second round of liposome-ELISA and FSEC (Fig. 5b). Finally, 2 stable hybridoma cell lines (clone #43 and clone #55) were established. The denatured dot blot analysis showed that IgG#43 recognized the native conformation of AdipoR1, whereas IgG#55 recognized the linear epitope (Fig. 5c). In addition, ELISA and FSEC analyses revealed that both IgG#43 and IgG#55 cross-reacted with AdipoR2Δ99 (data not shown). The cDNAs encoding the V_H_ and V_L_ regions of IgG#43 were cloned from hybridoma cells, according to the standard method [18]. The very N-terminal amino acid residues of the V_H_ and V_L_ fragments were determined by Edman degradation, and the 15 residue sequences of the N-termini of the V_H_ and V_L_ regions from clone #43 were determined as EVLLQQSGPELVKPG and DIQMTQSPASLSASV, respectively. The base sequences of the cloned cDNAs were corrected accordingly. The variable region of IgG#43 (Fv43) was synthesized by the *E. coli* cell-free protein synthesis method, and purified to homogeneity by column chromatography. The yield of the purified Fv43 was 0.3 mg per 1 ml cell-free synthesis reaction.

### Data collection and processing

**Fig. 6:**
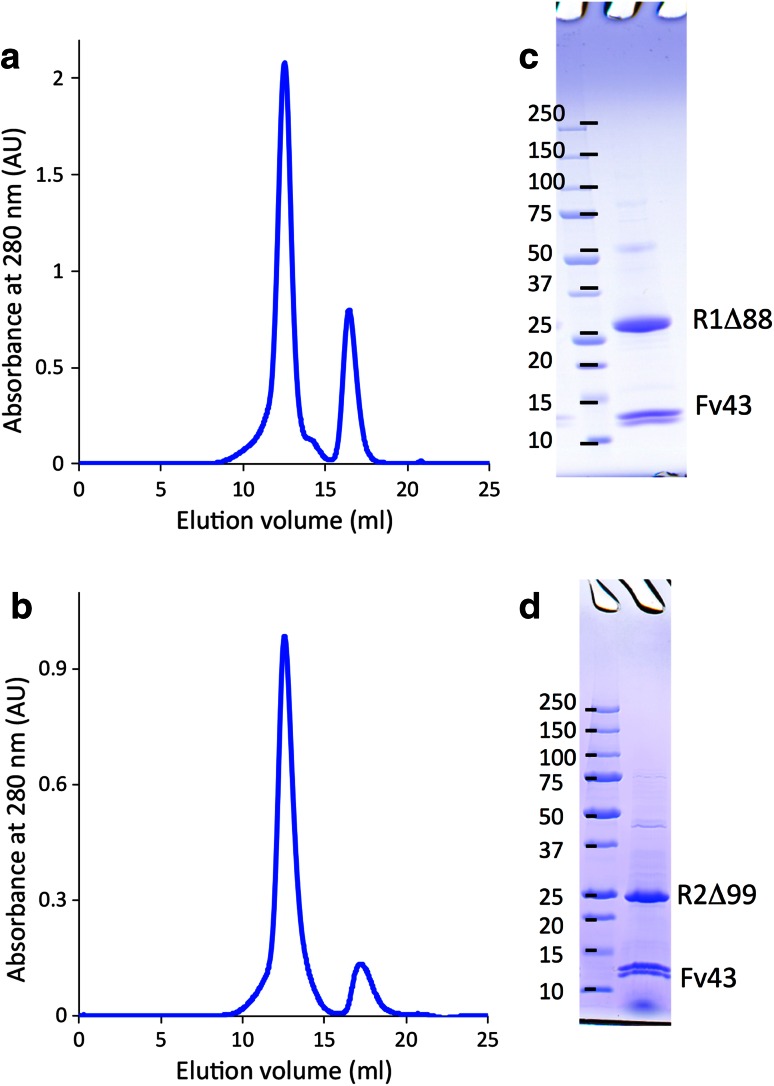
Isolation of the AdipoR1Δ88·Fv43 and AdipoR2∆99·Fv43 complexes. **a**, **b** Gel filtration chromatograms of the AdipoR1Δ88·Fv43 (**a**) and AdipoR2∆99·Fv43 (**b**) complexes. **c**, **d** SDS-PAGE analysis of the crystallization samples of the AdipoR1Δ88·Fv43 (**c**) and AdipoR2∆99·Fv43 (**d**) complexes

**Fig. 7:**
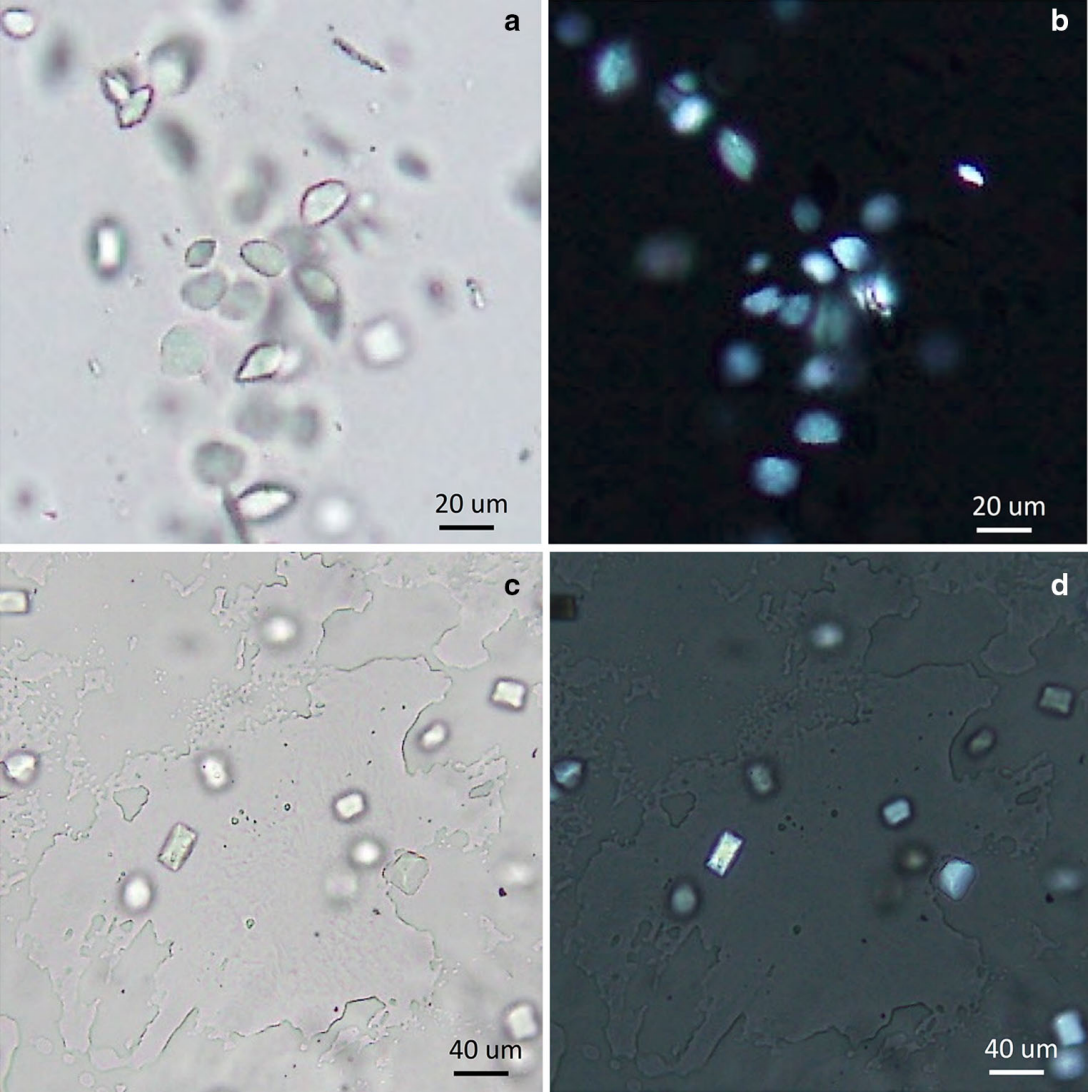
Crystals of the FLAG-tagged AdipoR1Δ88·Fv43 and AdipoR2∆99·Fv43 complexes. **a**, **b** Crystals of the FLAG-tagged AdipoR1Δ88·Fv43 complex. **c**, **d** Crystals of the AdipoR2∆99·Fv43 complex. Crystals are shown in bright field (**a**, **c**) and under crossed polarizers (**b**, **d**)

The cell-free produced Fv43 was mixed with the purified AdipoR1Δ88 and AdipoR2Δ99, and the AdipoR1Δ88·Fv43 and AdipoR2Δ99·Fv43 complexes were isolated by gel filtration chromatography (Fig. 6). The AdipoR1Δ88·Fv43 and AdipoR2Δ99·Fv43 complexes were crystallized by the lipidic mesophase method. The AdipoR1Δ88·Fv43 crystals with 10–15 μm lengths were obtained in 100 mM bicine buffer (pH 8.0) containing 50–150 mM MgSO_4_ and 29–33 % (v/v) PEG400 (Fig. 7a, b). The AdipoR2Δ99·Fv43 crystals with 30–40 μm lengths were obtained in 100 mM Na-citrate buffer (pH 6.0) containing 375–425 mM K-citrate and 28–30 % (v/v) PEG400 (Fig. 7c, d). The crystals reached their full sizes within one week.

**Fig. 8:**
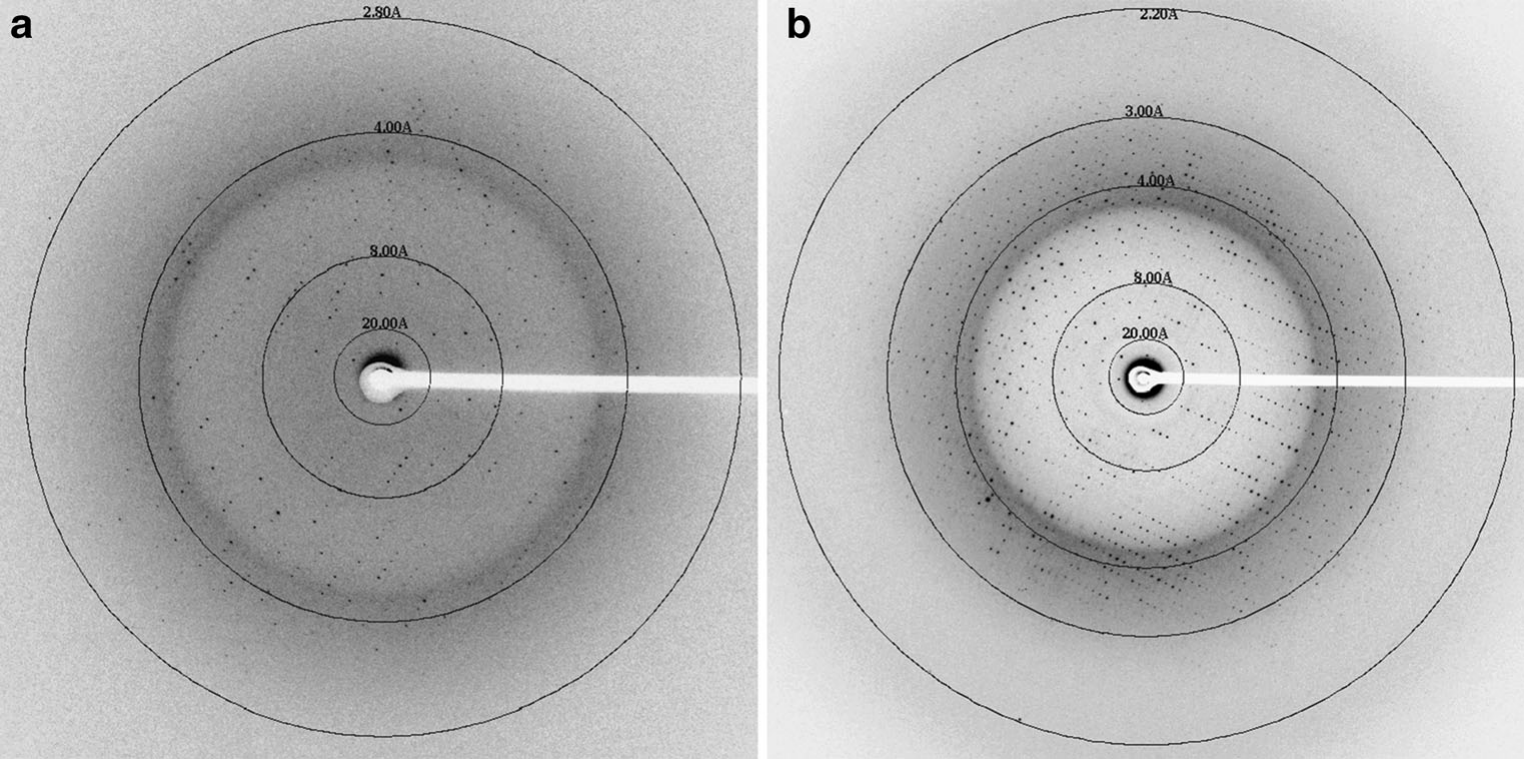
X-ray diffraction images of the AdipoR1Δ88·Fv43 complex (**a**) and the AdipoR2∆99·Fv43 complex (**b**)

We successfully collected all of the data sets by the helical scan method, with a microbeam of 1 μm × 10 μm (horizontal × vertical) at beamline BL32XU at SPring-8 [24–26]. The AdipoR1Δ88·Fv43 and AdipoR2Δ99·Fv43 crystals diffracted up to 2.8 and 2.2 Å resolutions, respectively (Fig. 8). Data collection from the AdipoR1 crystal was limited to 10–30 images per crystal, due to radiation damage in the microcrystals, and therefore the data from six crystals were merged to complete the data set. The diffraction data of the AdipoR2 crystal were collected from a single crystal. The data collection statistics are shown in Table 1. The AdipoR1Δ88·Fv43 and AdipoR2Δ99·Fv43 crystals belonged to the space groups *C*222_1_, with unit cell parameters *a* = 92.7, *b* = 194.4, *c* = 74.4 Å, and *P*2_1_2_1_2, with unit cell parameters *a* = 74.6, *b* = 108.6, *c* = 101.0 Å, respectively. Under the assumption that the asymmetric unit contained one AdipoR1Δ88·Fv43 complex or one AdipoR2Δ99·Fv43 complex, the Matthews coefficients of the AdipoR1Δ88·Fv43 and AdipoR2Δ99·Fv43 complexes were calculated to be 2.8 Å^3 ^Da^−1^ with a solvent content of 56.1 % and 3.6 Å^3^Da^−1^ with a solvent content of 65.4 %, respectively. An initial phase for the AdipoR2Δ99·Fv43 complex was obtained by molecular replacement using the Fv fragment (the V_H_ and V_L_ fragments from PDB IDs 1E6J and 1FDL, respectively) in Phaser [37] as search models. The refinement is in progress.

**Table 1:** Data collection statistics

Structure	AdipoR1Δ88·Fv43 complex	AdipoR2Δ99·Fv43 complex
No. of crystals	6	1
X-ray source	BL32XU, SPring-8	BL32XU, SPring-8
Wavelength (Å)	1	1
Space group	*C*222_1_	*P*2_1_2_1_2
Cell dimensions		
*a*, *b*, *c* (Å)	92.7, 194.4, 74.4	74.6, 108.6, 101.0
*α*, *β*, *γ* (°)	90.0, 90.0, 90.0	90.0, 90.0, 90.0
No. of reflections measured	135111	145165
No. of unique reflections	16509	32174
Resolution (Å)	20.00–2.80 (2.90–2.80)	19.52–2.40 (2.49–2.40)
*R*_merge_	0.176 (>1)	0.115 (1.297)
Mean *I*/σ(*I*)	8.5 (1.3)	8.6 (1.2)
Completeness (%)	97.2 (99.1)	98.3 (99.4)
Redundancy	8.2 (8.0)	4.5 (4.5)
V_M_ (Å^3^ Da^−1^)	2.8	3.6
Solvent content (%)	56.1	65.4

Values in parentheses are for the outer shell
